# Awake Craniotomy for the Excision of a Pediatric Cerebral Arteriovenous Malformation for Language Preservation: A Case Description

**DOI:** 10.3390/jpm15070319

**Published:** 2025-07-15

**Authors:** Melody Long, C. Thiaghu, Tien Meng Cheong, Ramez W. Kirollos, Julian Han, Lee Ping Ng, Sharon Y. Y. Low

**Affiliations:** 1 Department of Pediatric Anesthesia, KK Women’s and Children’s Hospital, 100 Bukit Timah Road, Singapore 229899, Singapore; 2 Department of Anesthesiology, Intensive Care and Pain Medicine, Tan Tock Seng Hospital, 11 Jalan Tan Tock Seng, Singapore 308433, Singapore; 3 Department of Neurosurgery, National Neuroscience Institute, 11 Jalan Tan Tock Seng, Singapore 308433, Singapore; 4 Neurosurgical Service, KK Women’s and Children’s Hospital, 100 Bukit Timah Road, Singapore 229899, Singapore; 5 SingHealth Duke-NUS Neuroscience Academic Clinical Program, 11 Jalan Tan Tock Seng, Singapore 308433, Singapore; 6 SingHealth Duke-NUS Pediatrics Academic Clinical Program, 100 Bukit Timah Road, Singapore 229899, Singapore

**Keywords:** awake craniotomy, pediatric cerebral arteriovenous malformation, awake language mapping, processed EEG

## Abstract

**Background:** Awake craniotomy (AC) surgeries are less common in the pediatric population in comparison to their adult counterparts. Nonetheless, they can be considered for selected cases whereby speech preservation is paramount during maximal safe resection of intracranial lesions. We describe a case of AC for the excision of a brain arteriovenous malformation (bAVM) with language mapping in a pediatric patient. **Methods**: A previously well 16-year-old male presented with a spontaneous left frontal intracranial hemorrhage. Neuroimaging confirmed the cause to be a left antero-temporal bAVM centered in the insula. A decision was made for AC bAVM excision with language mapping for speech preservation. **Results**: As part of the pre-operative preparation, the patient and his caregivers were reviewed by a multidisciplinary team. For the conduct of the AC, the asleep–awake–asleep technique was used with processed EEG to guide anesthesia management. Additional modifications to make the patient comfortable included the avoidance of rigid cranial skull pins, urinary catheterization and central line insertion at the start of the surgery. **Conclusions**: Our experience concurs with the evidence that AC in children is a feasible option for select individuals. To our knowledge, this is the first detailed case description of a pediatric patient undergoing AC with language mapping for a bAVM. Emphases include a strong rapport between the patient and the managing multidisciplinary team, flexibility to adjust conventional workflows and limitations of neuroimaging adjuncts.

## 1. Introduction

Brain arteriovenous malformation (bAVM) is characterized by an arteriovenous shunt without the normal interposed capillary beds but with the presence of an arterial nidus [1,2]. In contrast to their adult counterparts, pediatric bAVMs have distinct clinical features with a higher proportion of ruptured presentations [3,4], whereby the most common cause of spontaneous intracranial hemorrhage (ICH) in children is bAVM rupture [2]. Regardless of age, ICH from bAVMs is a significant source of neurological morbidity and mortality [5,6]. Ultimately, the primary aim of bAVM treatment is to completely obliterate the lesion to prevent bleeding and further re-bleeding and/or to alleviate any clinical symptoms attributed to the bAVM [7,8]. This is especially relevant in children, given their extended life expectancy and higher cumulative risk of rupture compared to adults [9,10,11]. In addition, some studies have shown that children have better long-term outcomes after microsurgical resection of bAVMs [7,12]. Nonetheless, we are cognizant that invasive surgery may pose higher risks of post-operative functional deficits for bAVMs located in the eloquent cortex.

Separately, awake craniotomy (AC) with brain mapping is an established technique for functional preservation in adult neuro-oncology, especially for glioma surgery [13,14]. This patient-centric approach allows the neurosurgeon to maximize lesion resection while preserving neurological function. A common indication for AC is surgery in the eloquent language cortex. Advancements in neuroimaging techniques such as diffusion tensor imaging (DTI) that generate white matter tractography have shown to provide insights into the structural connectivity of language pathways in the brain [15]. However, there are studies that report that radiology alone may not be sufficiently sensitive to delineate the patient’s actual functional topography [16,17,18]. Put together, an awake patient can provide invaluable inputs to help the operating neurosurgeon know in real time the extent the lesion can be excised while minimizing the risk of neurological injury to a vital region of the brain [19,20,21]. Despite its purported success in adults, the role of AC in the pediatric population remains undefined. Cited reasons include uncertainty surrounding their ability to tolerate the discomfort, heightened anxiety associated with intraoperative mapping, live monitoring and an overall poorly defined patient selection criteria [20,21].

At the time of this writing, the use of AC for language mapping in a pediatric bAVM patient has not been previously detailed in the literature. We therein report such a case and discuss its nuances in corroboration with contemporary publications.

## 2. Case Report

**Figure 1:**
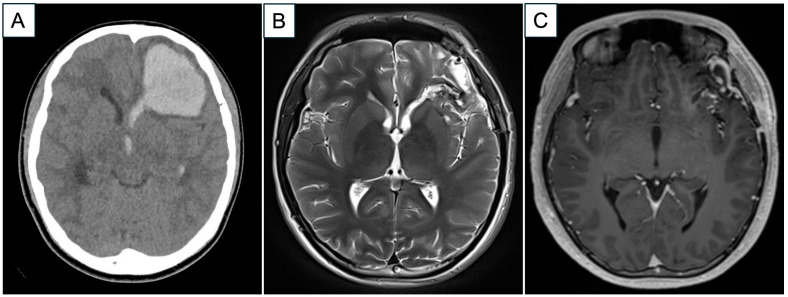
(**A**) Representative CT image in axial direction depicting a large, acute left frontal hematoma with intra-ventricular extension. (**B**) Representative MRI axial image in T2-weighted sequence post-ICH evacuation. There is improvement in the local mass effect. (**C**) Representative post-contrast T1-weighted MRI axial image that shows a likely vascular malformation in the left antero-temporal region. (Abbreviations: CT = computed tomography, ICH = intracerebral hematoma, MRI = magnetic resonance imaging).

A previously well 16-year-old right-handed male presented with a sudden onset of acute neurological deterioration and a unilaterally dilated left pupil. An urgent computed tomographic (CT) brain scan showed a large spontaneous left frontotemporal ICH with mass effect and midline shift. He underwent an emergency left craniotomy and evacuation of the ICH for life-saving measures. Follow-up magnetic resonance imaging with angiography sequences (MRI/MRA) reported a left bAVM (Spetzler–Martin Grade 2) centered in the left frontal operculum, involving the insula (Figure 1).

**Figure 2:**
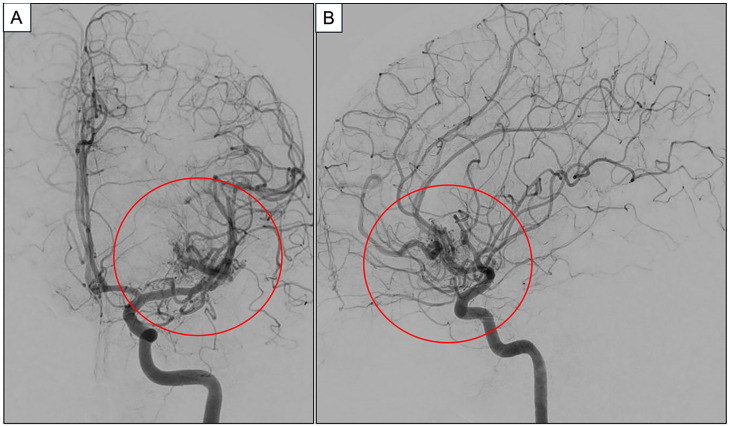
Representative images from cerebral DSA’s left internal carotid arterial phase run in (**A**) anteroposterior and (**B**) sagittal views, respectively. A left anterior temporal bAVM centered in the left operculam-insular region with a diffuse nidus is featured in both images (red circle). (Abbreviations: bAVM = brain arteriovenous malformation, DSA = digital subtracted angiography).

This finding was further delineated with a digital subtracted cerebral angiogram (DSA). Detailed angioarchitecture revealed the bAVM to have feeding arteries arising from the orbitofrontal and prefrontal branches of the left middle cerebral artery (M3 and M4). The nidus was approximately 2 cm but diffuse in nature. It drained into smaller veins, which subsequently united into a large frontal vein, before draining into the anterior superior sagittal sinus (Figure 2).

**Figure 3:**
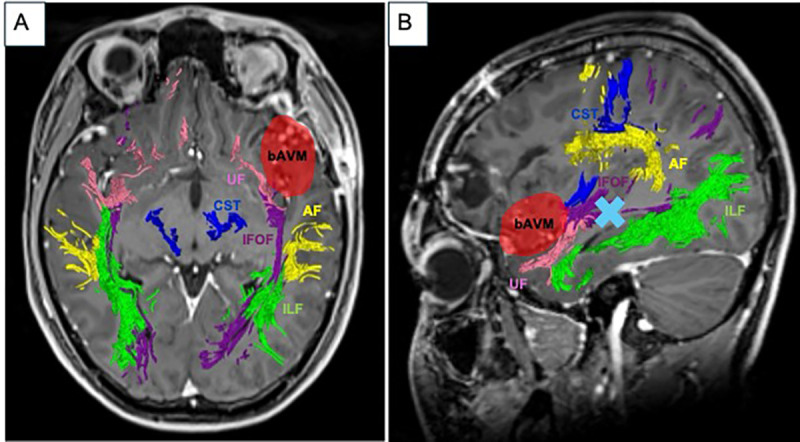
Representative post-contrast T1-weighted MRI brain images fused with post-processed DTI tractography in relation to the bAVM region (red) using Modus Plan (Synaptive Medical, Toronto, Canada) in (**A**) axial and (**B**) sagittal directions. The arcuate fasiculus (AF) is noted to be posterior to the bAVM. Of note, the light blue arrow in (**B**) depicts the region whereby direct cortical stimulation was applied and the patient demonstrated speech apraxia. (Abbreviations: AF = arcuate fasiculus, bAVM = brain arteriovenous malformation, CST = corticospinal tract, IFOF = inferior fronto-occipital fasiculus, ILF = inferior longitudinal fasiculus, UF = uncinate fasiculus).

After the ICH evacuation, the patient gradually recovered back to his baseline neurological function after a short period of inpatient neurorehabilitation. Upon discharge, he returned to school to complete the remaining segment of his semester. In the subsequent weeks, updated neuroimaging was arranged as part of the next steps of his management. This included an MRI with diffusion tensor imaging (DTI) sequences. Of note, post-processed white matter tractography demonstrated the proximity of the left uncinate fasiculus to the bAVM nidus (Modus Plan, Synaptive Medical, Toronto, ON, Canada) (Figure 3).

During a follow-up outpatient review, various options for the management of his bAVM were discussed at length with the patient and his caregivers. These included microsurgical resection, stereotactic radiosurgery and conservative surveillance. Pertaining to surgery, the option of AC to excise the bAVM was presented in view of the risk of post-operative speech deficit, as it was adjacent to the uncinate fasciculus. Under such circumstances, the primary concern was that intraoperative white matter dissection may unintentionally cause a speech deficit. After a period of deliberation, the patient decided to proceed with AC excision of his bAVM with language mapping. As part of the pre-operative preparation, the patient was reviewed in the anesthesia clinic with his caregivers. He was shown videos of the designated operating room’s infrastructure and internal set-up and given opportunities to clarify the process. Separate neuropsychological assessments of his developmental ability to tolerate the procedure and baseline speech responses to a series of picture-naming cards were also practiced as part of the preparation.

Pertaining to the actual surgery, the “asleep–awake–asleep” technique was applied for the conduct of the AC. Briefly, standard American Society of Anesthesiologists (ASA) monitors were placed. Concurrently, peripheral intravenous cannulas, an arterial line and central venous catheter were sited. An adhesive SedLine^®^ brain monitoring sensor was placed on his forehead in the conventional position, ensuring the cord did not interfere with the surgical field. The patient’s head was placed on a horseshoe without rigid pin fixation. To reduce discomfort from the craniotomy incision, a bilateral scalp block with Bupivacaine 0.5% was performed prior to skin incision. Following that, the total intravenous anesthesia (TIVA) technique using target-controlled infusion (TCI) propofol (Eleveld model) [22] was used, along with a remifentanil infusion. The patient’s depth of anesthesia was monitored and adjusted based on the following real-time parameters: spectral edge frequencies (SEFs), Density Spectral Array (DSA) and concurrent EEG waveforms. For anesthetic induction, intravenous (IV) propofol was started at CeT 2.5 mcg/mL (Eleveld model) and titrated to 3.5 mcg/mL, with IV remifentanil 0.15 mcg/kg/min and IV Fentanyl 50 mcg. A size 3 supraglottic airway (SGA) was inserted when the SEF was in the range of 5 to 10 whilst maintaining spontaneous ventilation. IV paracetamol 15 mg/kg was given for analgesia in addition to the scalp block that was performed pre-incision. Anesthesia was titrated to maintain SEF 10 to 15 during the asleep phase, with CeT propofol between 3.2 and 3.5 mcg/mL. On the DSA, red bands were seen in the alpha (8 to 12 Hz) and delta (0 to 4 Hz) frequencies, reflecting the spectrogram signature of a propofol-based anesthetic. During intraoperative language testing, propofol and remifentanil were stopped to achieve an SEF greater than 20 during the awake phase, at which point the SGA was removed [23]. On the DSA, a “zipper opening” pattern was observed when the propofol was stopped. This was due to a gradual shift from slow and alpha oscillations towards higher-frequency gamma and beta oscillations [24]. The decrease in amplitude of oscillations is reflected with the red bands turning yellow. For our patient, approximately 30 min was required after stopping propofol before he was able to cooperate with the language testing.

**Figure 4:**
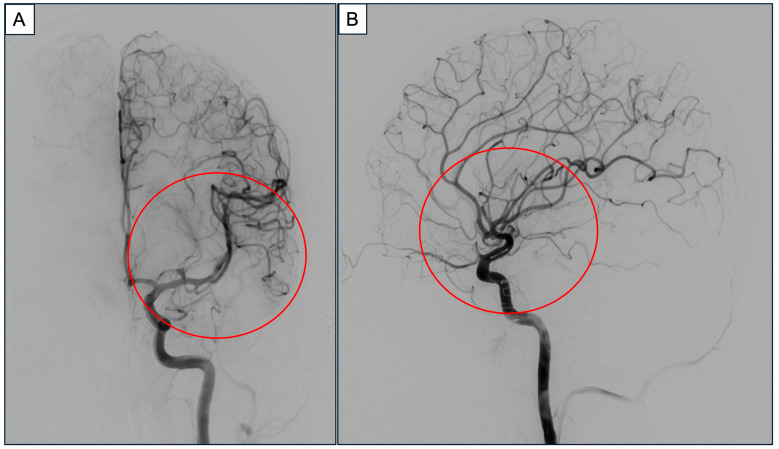
Representative images from cerebral DSA’s left internal carotid arterial phase run after the bAVM excision in (**A**) anteroposterior and (**B**) sagittal views, respectively. The bAVM was excised without any residual nidus (red circle). (Abbreviations: bAVM = brain arteriovenous malformation, DSA = digital subtracted angiography).

**Figure 5:**
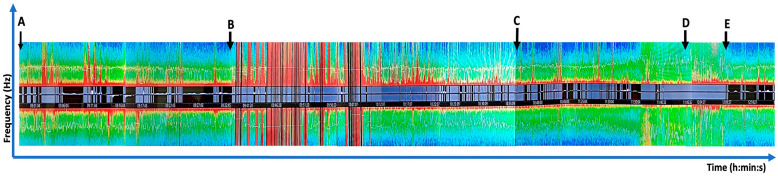
Chronological illustration of Density Spectral Array data from processed EEG across various time points in the surgery. Red/orange represents higher power in a particular frequency range. “Zipper opening” pattern seen between points C and D representing a more awake state. *y*-axis, frequency (Hz); *x* axis, time represented as hours: minutes: seconds (h:min:s). Specified timepoints as per the following: A = supraglottic airway insertion; B = knife-to-skin commenced; C = propofol infusion stopped; D = patient awakened for language mapping; E = patient re-anaesthetized for completion of the surgery.

The bAVM was located in the deep frontal opercular region and was primarily fed by cortical M3 and M4 branches of the left middle cerebral artery. A cortisectomy through the left frontal operculum was required to access the lesion. Neuronavigation was initially used to identify cortical regions whereby operative trajectories into the bAVM nidus could potentially be approached. Next, language mapping was performed by direct cortical electrical stimulation and electrocorticography. Here, the standard Penfield method via a hand-held monopolar ball tip probe was used [25,26]. A monophasic pulse duration of 1.0 milliseconds starting at a frequency of 30 Hz was applied for approximately 3 s at each site. This stimulation started at a low intensity of 1.0 mA, which was gradually increased as needed until a speech arrest/apraxia was encountered. Here, intraoperative awake language mapping was instrumental in identifying the non-eloquent cortex within the left frontal opercular area, which served as a safe entry point. Upon electrode stimulation of a cortical region involving the posterior aspect of the superior temporal gyrus, he demonstrated verbal apraxia. A marker was placed on the area of interest and avoided. An alternative surgical trajectory was planned anterior to the marker whereby both cortical and subcortical stimulation did not cause any speech anomality. A generous cortisectomy through the inferior frontal gyrus, anterior to the identified speech area, was performed to create a broad operative corridor. This allowed a wide circumferential dissection around the bAVM nidus while preserving the superficial draining vein. Subcortical mapping during the parenchymal dissection facilitated the identification and preservation of eloquent subcortical pathways, specifically the anterior portion of the arcuate fasciculus, a component of the dorsal speech pathway. The remainder of the language testing period was uneventful. The patient remained calm and cooperative during the testing period of 25 min despite not being on any propofol or remifentanil. He successfully completed the brain mapping with speech testing before being re-anaesthetized and intubated for the conclusion of the procedure. Intraoperative findings confirmed that the bAVM had a diffuse architecture, which is associated with a higher risk of recurrence, particularly in the pediatric population [27,28]. Under such circumstances, our approach in adopting a wider resection margin is especially important to facilitate the identification and control of feeding arteries and reduces the risk of residual nidus, thereby lowering the potential for recurrence. The post-resection DSA demonstrated no residual bAVM (Figure 4). At the end of the surgery, he was extubated with intact speech and neurological function. Figure 5 shows the chronological sequence of events, correlated to the Density Spectral Array.

The remainder of his inpatient stay was otherwise uneventful. In the ward, the patient was re-assessed by the speech therapist who confirmed that his pre-operative baseline was maintained. Upon discharge, he was well and neurologically intact. A surveillance MRI/MRA brain scan performed approximately 6 months after his surgery reported no residual vascular lesion.

## 3. Discussion

In this era of modern neurosurgery, AC is an established approach for lesion resection in eloquent brain regions. Broadly speaking, eloquence is typically referred to as the cortical areas assigned to a specific function, essentially the motor areas and the areas of language and visual function, in addition to the brain stem and the deep structures. Indisputably, the AC procedure requires patients to remain calm, cooperative and communicative during the awake brain mapping part of the surgery [29]. Under such circumstances, the pediatric population poses specific challenges towards implementation of AC due to wide variability in patients’ cognitive development, level of maturity and willingness to cooperate [30,31]. Awake craniotomies in children, though infrequent, can be effective when patient selection is executed meticulously [21]. To date, most publications on AC in the pediatric population are limited to case reports and small case series [20,32,33,34]. Nonetheless, two recent meta-analyses focused on AC in children demonstrate that the procedure is feasible with few complications [20,21]. Overall, the premature termination of the awake phase is observed to be low, and the most common reasons were intraoperative seizures, excessive fear and pain in this affected cohort [20,21]. One noteworthy observation is that the minimal age for pediatric AC is not established, and there are no defined guidelines on the criteria for patient selection in this age group [35]. Some studies performed a conscientious identification of the individual’s level of cognitive development and correlated assessment pre-operatively by a neuropsychologist [35,36,37]. Another described method is to employ a simulated operating theatre experience to evaluate the child’s eligibility [35]. At the time of this writing, there are only two previous reports of AC for pediatric bAVM in the literature [21,34]. We postulate that the lack of these cases is likely due first to bAVMs being a rare entity and next that the perioperative bleeding risks of bAVM are high, with potentially devastating sequalae [7].

Our team applied the TCI Eleveld model, which is suitable for a broad population range as an effective means of administering propofol [38]. It is especially useful in this situation, where precise titration of the CeT to the SEF values is critical to accurately predict the intraoperative “wake up period” to facilitate brain mapping. In congruency with the contemporary literature, our experience with processed EEG application is helpful in optimizing anesthetic management and allowing for precise titration of depth in accordance with the SEF, DSA and raw EEG waveforms [23]. Currently, the usage of processed EEG is not commonplace in AC procedures due to concerns of the monitoring strip placement on the forehead interfering with the surgical field and/or the initial registration segment of the neuronavigation set-up. However, as described in our case, we demonstrate that it can be used effectively to facilitate communication between the anesthetist and neurosurgeon regarding readiness for speech assessment [39,40]. While standard placement of the EEG sensor is preferred, alternative locations may be considered if necessary, though they may present varying degrees of accuracy in the depth of anesthesia [41].

For the excision of intrinsic lesions involving the dominant eloquent cortex, direct cortical and subcortical bipolar electrostimulation during AC is now recognized as the gold-standard approach [42]. Nonetheless, a subset of patients is unable to tolerate awake brain surgery [43]. For those who require monitoring of the motor tract, the use of transcranial high-frequency repetitive electrical stimulation under general anesthesia is a feasible alternative [44]. Not unexpectedly, there are no such proxy techniques for language mapping. This is because human speech is a complex process involving several integrated intracranial components. Here, several white matter tracts are crucial for speech, including the arcuate fasciculus (AF), the uncinate fasciculus (UF), the inferior fronto-occipital fasciculus (IFOF), and the frontal aslant tract (FAT). In particular, the AF is an important connection between the temporal and frontal language areas, facilitating phonological production and semantic processing [45,46]. In addition, the posterior supramarginal gyrus (SMG) has been shown to produce performance errors (such as slurred speech, stuttering and so forth) during cortical stimulation, while the posterior middle temporal gyrus often generates semantic paraphasia during stimulation. These deficits are in tandem with the dorsal and ventral pathways of language processing, whereby stimulation above the superior temporal sulcus frequently impairs phonological processing while in inferior temporal sites it frequently impairs semantic processing [47]. Building on the above, the most common intraoperative language mapping task employed is, therefore, picture naming [13]. To delineate eloquent areas that may be involved by the lesion of interest, electrostimulation of different cortical regions is usually applied to produce various speech errors [13].

Intraoperatively, defining the limits between what is eloquent versus what is not eloquent may not be so straightforward. In contrast to most intrinsic brain lesions, bAVMs are vascular anomalies that alter cerebral perfusion and cortical organization in the developing brain. These changes can result in language, motor, and/or visual function shifts within gyri or even to the contralateral hemisphere from its expected neuroanatomical position [48,49,50]. This is especially applicable in our case, whereby the bAVM nidus is diffuse. For such cases, the bAVM boundaries with the surrounding normal tissue are difficult to determine, and incomplete resection is often a concern [51]. In addition, a diffuse nidus is associated with higher risks of rupture during surgery [51]. Although neuroimaging adjuncts such as MRI DTI facilitate the understanding of cortical areas affected by the bAVM, there are still pitfalls. Examples include a low signal-to-noise ratio, susceptibility to artifacts and difficulty in accurately depicting fiber crossings. Furthermore, the lack of a standardized analysis and reliable post-processing protocols contribute to challenges in the reproducibility and accuracy of DTI tractography [52]. Although DTI sets boundaries for the resection of lesions located near the AF, post-processed tractography does not always prevent a post-operative speech deficit [53,54]. This is because clinical studies have demonstrated that the Broca and Wernicke areas tend to vary among individuals, thus precluding the accuracy of what may be conceived to be the functionally active AF in DTI images [54]. To date, these limitations seem to be the main drawback of DTI fiber tracking of AF [54]. Under such circumstances, the use of AC is a logical approach to improving patient safety in terms of preserving speech functions [54,55]. Another established modality for imaging brain activity is functional MRI (fMRI)—this has the ability to map out the spatiotemporal distribution of neuronal activation either in a resting state or in response to stimuli [56]. Generally, bAVMs are characterized by direct connections between arteries and veins, which tend to steal blood flow from surrounding brain tissue [7,57]. Therefore, these innate hemodynamic differences between the bAVM and adjacent normal parenchyma may alter blood oxygen level-dependent (BOLD) signaling, hence leading to inaccurate functional mapping [48,56,58]. On a separate note, direct cortical electrical stimulation is often cited as an established method to map the eloquent cortex, especially when there are concerns of overlap with surgical margins [26]. Nonetheless, the operating team has to be cognizant of the associated risks of current-related neural injury, provoked seizures and inter-individual variations in specific localizations of the human cortex [59]. To mitigate this in our patient, the consensus was to start with more conservative parameters for the stimulation in comparison to the adult literature [25,26].

To enable the AC experience to be less challenging for the patient, the following modifications were implemented after a detailed multidisciplinary team discussion. Firstly, we avoided rigid cranial pinning to alleviate feelings of discomfort and anxiety associated with the use of the Mayfield skull frame [60,61]. This phenomenon has been previously reported in the literature by some patients who express discomfort and anxiety attributed to the awake phase of the surgery [60,62]. However, the caveat is that in the event the patient becomes disoriented, there is risk of more freedom of movement on the operating table, requiring the neurosurgeons to manually restrain them [61]. Also, rigid cranial fixation may pose airway problems during awake surgery and hinder airway access for the anesthetic team. A deeper level of sedation may also be necessary to ensure patient comfort, which can cause respiratory and airway issues and affect communication with the patient during the awake phase [63]. Additionally, we delayed the insertion of the indwelling urinary and central venous catheters until the language mapping was over and the patient was intubated. Put together, we believe these variations, albeit unconventional, contributed to easing the patient’s overall anxiety for the procedure.

## 4. Conclusions

Optimal management of pediatric bAVM is challenging because extended, long-term outcomes are paramount in this age group. To our knowledge, this is the first case description of a pediatric patient undergoing AC with language mapping for a bAVM. Of note, we report that processed EEG aids in optimizing anesthetic management, allowing for precise titration of depth and facilitating effective communication between the anesthetist and neurosurgeon regarding key moments for speech assessment. Overall, our experience concurs with the published literature that AC in children is a feasible option for select individuals. Important highlights include the following: firstly, the role of a multidisciplinary healthcare team working cohesively with the patient and caregivers; next, the advantages of flexibility to change conventional workflows for patient-centric situations; and, finally, the need to understand the limitations of neuroimaging adjuncts specific to the procedure of choice. In the current setting of evidence-based medicine, we advocate AC for suitable pediatric patients to preserve language function.

## Data Availability

The original contributions presented in this study are included in the article. Further inquiries can be directed to the corresponding author.
